# Evidence of Influence of Genomic DNA Sequence on Human X Chromosome Inactivation

**DOI:** 10.1371/journal.pcbi.0020113

**Published:** 2006-09-01

**Authors:** Zhong Wang, Huntington F Willard, Sayan Mukherjee, Terrence S Furey

**Affiliations:** Institute for Genome Sciences and Policy, Duke University, Durham, North Carolina, United States of America; Fred Hutchinson Cancer Research Center, United States of America

## Abstract

A significant number of human X-linked genes escape X chromosome inactivation and are thus expressed from both the active and inactive X chromosomes. The basis for escape from inactivation and the potential role of the X chromosome primary DNA sequence in determining a gene's X inactivation status is unclear. Using a combination of the X chromosome sequence and a comprehensive X inactivation profile of more than 600 genes, two independent yet complementary approaches were used to systematically investigate the relationship between X inactivation and DNA sequence features. First, statistical analyses revealed that a number of repeat features, including long interspersed nuclear element (LINE) and mammalian-wide interspersed repeat repetitive elements, are significantly enriched in regions surrounding transcription start sites of genes that are subject to inactivation, while Alu repetitive elements and short motifs containing ACG/CGT are significantly enriched in those that escape inactivation. Second, linear support vector machine classifiers constructed using primary DNA sequence features were used to correctly predict the X inactivation status for >80% of all X-linked genes. We further identified a small set of features that are important for accurate classification, among which LINE-1 and LINE-2 content show the greatest individual discriminatory power. Finally, as few as 12 features can be used for accurate support vector machine classification. Taken together, these results suggest that features of the underlying primary DNA sequence of the human X chromosome may influence the spreading and/or maintenance of X inactivation.

## Synopsis

Female mammals have two X chromosomes while males have one X and one Y chromosome. To equalize dosage of X chromosome genes in males and females, one X in female cells is inactivated, repressing the expression of most genes on the chromosome. Despite the chromosome-wide nature of X inactivation, at least 10%–15% of genes “escape” this inactivation in human females and are still expressed on the inactivated X. Whether a gene escapes or is subject to inactivation is thought to be determined epigenetically, and it is unknown to what extent, if at all, the underlying genomic DNA sequence of the chromosome plays a role. In this work, the authors show that the DNA sequence surrounding genes that escape inactivation is significantly different from the sequence surrounding genes that are subject to inactivation. In fact, a small number of DNA sequence features can be used to predict with high accuracy whether a gene will escape or be subject to this silencing. This establishes strong evidence that epigenetic regulation is, at least in part, dependent on genomic sequence and organization and provides a list of candidate sequence features whose role(s) in X inactivation can now be explored.

## Introduction

X-chromosome inactivation suppresses gene expression on one of the two X chromosomes in female mammals to achieve dosage compensation between males and females ([1]; reviewed in [2,3]). During early stages of embryo development, one X chromosome is randomly chosen for inactivation. The chosen chromosome stably transcribes the *XIST* gene in the X inactivation center [4] to produce a noncoding RNA that has been shown to mediate the initial inactivation [5–7]. In a process not well understood, *XIST* transcripts spread along the chromosome [8], leading to heterochromatin formation along the length of the X. Subsequent maintenance of inactivation is accomplished through a diverse set of epigenetic histone modifications and DNA methylation in an *XIST*-independent manner (reviewed in [2,9]).

Recent studies have shown that this transcriptional repression is not complete. In both humans and mice, some X-linked genes escape inactivation to varying degrees (reviewed in [10,11]). While only a few genes have been shown to escape inactivation in mice, a significant proportion of X-linked genes (estimated at about 15%) are actively transcribed from the otherwise inactive human X chromosome, as demonstrated in both human heterozygous fibroblasts and human/rodent somatic hybrid cells that contain only the inactive human X chromosome. An additional 10% of the genes show variable inactivation status among different females [12]. The sequence basis, if any, for determining X inactivation status is unclear.

Interestingly, the distribution of genes that escape inactivation on the human X chromosome is nonrandom [12], although it is unclear whether this reflects the evolutionary history or the sequence content of different regions of the X. The chromosome can be roughly divided into two regions [13–15]: an X-conserved region (XCR) that originated at least 170 million years ago and spans nearly all of the long arm (Xq) and a portion of the short arm (Xp), and a much smaller X-added region (XAR) that was added to the short arm of the ancestral X after X inactivation mechanisms had evolved [16]. Nearly all genes in the XCR are subject to inactivation [12]. In contrast, the XAR, encompassing ~47 Mb of DNA, is highly variable, with nearly half of the genes escaping inactivation. As genes that are subject to or escape inactivation are clustered within the XAR, the expression data indicate that X inactivation is controlled at a domain level rather than at the level of individual genes [12].

As described above, X inactivation involves both an initial inactivation and the ongoing maintenance of this inactivated state. The lack of the complete silencing of some genes might result from the failure in the initial spreading of inactivation along the chromosome, the failure in a subsequent maintenance step after the initial inactivation, or a combination of the two (reviewed in [11,17]). The observed dichotomy of X-linked genes with respect to X inactivation, particularly within the XAR, may be due to the presence or absence of necessary spreading and/or maintenance signals that may be contained within the primary DNA sequence of the X [15] or to a variable pattern of epigenetic modifications along the X [18]. The identity of these putative sequence elements is not yet known.

Gartler and Riggs hypothesized that there may exist “way stations” on the X chromosome that aid the spreading of X inactivation [19]. Subsequently, Lyon proposed that these *cis*-acting elements may be long interspersed nuclear element 1 (LINE 1, or L1) retrotransposons [20]. The latter hypothesis was initially based on results from fluorescence in situ hybridization experiments that showed an enrichment of L1s on the X chromosome as compared with autosomes. It was supported by an analysis of sequence near a small number of X-linked genes in which L1 elements were found to be enriched in the vicinity of genes that were subject to inactivation [21]. This conclusion, however, was not supported by a more recent comparative study [22]; instead, that study reported that mammalian-wide interspersed repeat (MIR) elements, a subfamily of short interspersed nuclear elements, and CpG islands were significantly depleted in regions that escape inactivation. A third study based on word frequency analysis found GATA simple repeats to be enriched in the initial 7.5 Mb of the X chromosome, a region where all genes escape X inactivation [23]. In the above three studies, the number of potential sequence features analyzed were limited. In addition, the first two surveyed only a limited number of genes, while the last did not consider the full complement of genes that have been found to escape X inactivation. Thus, a more comprehensive analysis of the relationship of these and other sequence features with X inactivation status, using a more complete set of X-linked genes whose X inactivation status has been determined [12], may help to resolve these conflicting and incomplete results.

In this study, we have focused on primary DNA sequence features in regions around the complete set of human genes of known X inactivation status to further understand their potential role as signals in the spread and/or maintenance of X inactivation. Using the accurate and nearly complete X-chromosome sequence [15], we first systematically analyzed the genomic environments of genes that either are consistently subject to or escape inactivation by comparing the distributions of DNA sequence features from 2-, 5-, 10-, 20-, 50-, and 100-kb windows surrounding their transcription start sites. These DNA sequence features include the content of all annotated repeat families and subfamilies and the distribution of all possible 3- and 5-base sequences. This comparison of a more complete list of 73 escaping and 375 subject genes [12] indicates that the most informative sequence features show their greatest divergence in larger windows (50 kb and 100 kb) rather than smaller windows, supporting the hypothesis that inactivation status is determined at a domain level.

Multiple sequence features may influence X inactivation in an interdependent fashion. Determining these factors and their possibly combinatorial nature thus presents a complex problem. Machine learning classifiers such as those based on the support vector machine (SVM) algorithm have been successfully used to shed light on many complex biological problems, particularly gene expression analysis using microarray expression and/or sequence data [24–29]. We found that the X inactivation status for more than 80% of genes can be correctly predicted by linear SVM classifiers constructed using a set of sequence features from 50-kb and 100-kb windows, suggesting that signals for the spreading and/or maintenance of X inactivation may be embedded within primary DNA sequence.

**Figure 1:**
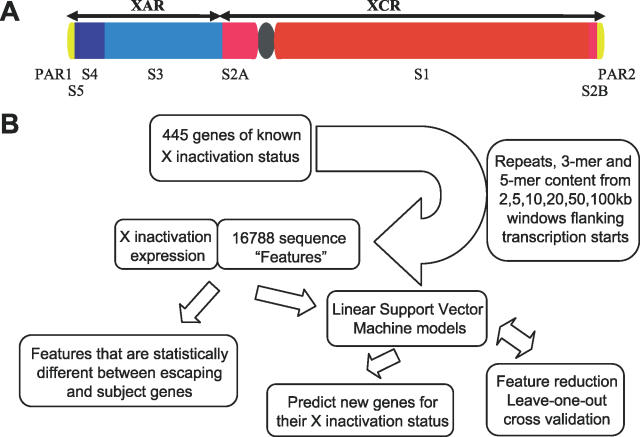
Data Analysis Strategy (A) A schematic drawing of the X chromosome delineating each evolutionary stratum [13,15]. (B) Strategy for statistical analysis and SVM training and classification.

Our statistical analysis and SVM classification experiments together, summarized in Figure 1, highlight a small set of sequence features that are both differentially enriched in regions escaping or subject to X inactivation and are important for accurate classification. This set includes the L1 and L2 LINE elements, a number of MIRs, and a few long terminal repeats (LTRs). In general, we found that severely reducing the number features does not affect classification accuracy. In fact, we found that as few as 12 features contain sufficient information for successful SVM classification. It is possible that some of these features may serve as signals for some step(s) involved in the spread and/or maintenance of X inactivation.

## Results

### The Genomic Contexts of Genes That Escape or Are Subject to X Inactivation Are Significantly Different

To investigate the relationship between X inactivation status and primary DNA sequence for a set of X-linked genes, we derived a set of 16,788 primary sequence features to represent the genomic context for each gene. To determine which features, if any, have different distributions in the genome sequence surrounding genes subject to inactivation as compared with those that escape inactivation [12], we initially performed modified Wilcoxon rank-sum tests (Materials and Methods). To assess the significance of the rank-sum values for each feature, we performed permutation analysis and calculated *q* values, a measure of false discovery rate [30]. We found 971 significant features at *q* < 0.02 and 2,345 features at *q* < 0.05 (Table S1).

We considered the possibility that these results may simply reflect the unique evolutionary history of the X chromosome rather than a specific relationship to X inactivation. It has been noted that many sequence characteristics, including GC content and repeat content are different between XAR and XCR [12,15]. To investigate whether sequence differences are solely due to evolutionary history, we first compared genes with different X inactivation statuses within XAR alone. Consistent with the above analysis using all genes, we found 1,506 significant features at *q* < 0.02 and 3,336 at *q* < 0.05 (Table S1). In addition, the features found to be differentially distributed in the XAR mirror those found to differ chromosome-wide as evidenced by an extremely high correlation (R^2^ = 0.74) between the rank-sum values calculated for the features in these two analyses.

To further explore a connection to evolutionary history, we analyzed the five unique strata that essentially separate the ancestral X chromosome sequence (XCR, strata 1–2) from the sequence added later (XAR, strata 3–5) [13,15] as shown in Figure 1A. Only stratum 3 contains a reasonable mix of both genes that escape (30 genes) and are subject (60 genes) to inactivation. The number of differentially distributed features and their significance are less compared with the previous two analyses with 100 significant features found at *q* < 0.15 and 449 at *q* < 0.20 (Table S1), but this does indicate that significant differences do exist. Again we see a high correlation between rank-sum values in this set compared with those in the XAR (R^2^ = 0.72) and in the whole chromosome (R^2^ = 0.55), suggesting that similar differences are being found in all three analyses.

From these data, we conclude that significant features commonly identified in these analyses most likely represent global differences between the genomic environment of escaping and subject genes and not simply regional differences (Table S2). Below we discuss the particular significant features in more detail. Overall, these results show that genes subject to and escaping inactivation have very different genomic contexts, suggesting that DNA sequence may underlie and/or contribute to the ability of X inactivation to silence gene transcription.

### Several Sequence Features Have Significantly Different Distributions in the Genomic Environments of Genes That Escape from or Are Subject to X Inactivation

Among those repeat sequence features found to have significantly different distributions chromosome-wide and in the XAR *(q <* 0.05), L1s and MIRs are among the most consistently enriched in regions surrounding the transcription starts of genes that are subject to X inactivation. In contrast, Alu elements are clearly the most consistently enriched in regions surrounding the transcription starts of genes that escape inactivation (Table S1). Furthermore, the distributions of these features show significant differences in multiple window sizes, especially 50-kb and 100-kb windows, located both upstream and downstream of the transcription start site (Table S1). These results suggest that the larger genomic environment, and not simply the promoter context, may be most relevant for determining X inactivation status.

Only ten repeat sequence features are significant chromosome-wide and in the XAR (*q* < 0.05) and also within stratum 3 (*q* < 0.2), shown in Table 1 and Table S2. As might be expected, we see L1, MIR, and Alu elements in this set and all features taken from 50-kb or 100-kb windows.

**Table 1:**
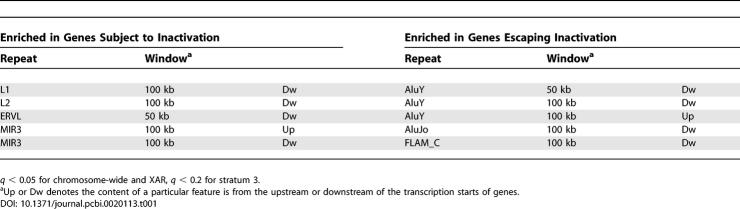
Repeat Features Whose Distributions are Significantly Different in All Three Evolutionary Stratifications when Comparing Genomic Environments of Genes Subject to and That Escape Inactivation

It has been reported that young L1 (subfamily L1Ps) members show greater enrichment on the X chromosome than older ones (subfamily L1Ms) [21], thus raising the possibility that L1Ps may be a better candidate for X inactivation signals. In our study, the content of both L1Ms and L1Ps were found to be significantly different when considering all escaping and subject genes (unpublished data), but neither of these is among those consistently enriched within the evolutionary based subdivisions we explored.

**Figure 2:**
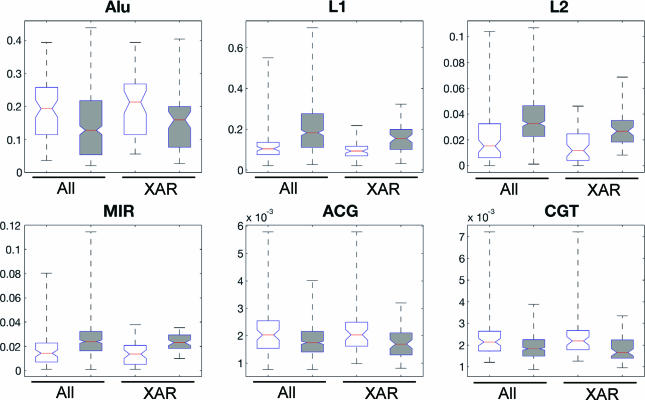
Boxplots Representing the Content of Alu, L1, L2, MIR, and ACG/CGT 3-mers in 100-kb Windows Surrounding the Transcription Starts of Genes For each boxplot, horizontal lines indicate the locations of the lower quartile, median, and upper quartile values. Notches represent a robust estimate of the uncertainty about the medians for box-to-box comparison. Boxes whose notches do not overlap indicate that the medians of the two groups differ at the 0.05 significance level. Clear boxes represent genes that escape inactivation, while shaded boxes represent genes subject to inactivation [12]. Analyses were done with all genes for which X inactivation status is known (*n* = 448) or with genes from the XAR region (*n* = 110). *y*-Axes in all plots represent the average content of each sequence feature in 100-kb windows surrounding the transcription starts of genes.

Our statistical analysis also revealed that the concentration of several 3-base and 5-base sequences (3-mers and 5-mers) is consistently different between the two classes of genes (Table S1). The association of these 3-mers and 5-mers with escaping or subject genes is primarily based on GC content. All of the 55 most significant 3-mers (*q* < 0.02, chromosome-wide) and 464 of the 515 5-mers (90.1%) that are enriched around escaping genes are GC rich, while all 27 3-mers and 275 of 316 5-mers (87.0%) enriched around subject genes are AT rich. Furthermore, 78.1% of 3-mers and 79.2% of 5-mers in regions around escaping genes contain a CG dinucleotide. Strikingly, among the short sequences that are very significantly enriched (*q* < 0.012) near genes that escape inactivation, the top 12 3-mers with respect to rank-sum values are CGT/ACG in various window sizes, while the top eight and 43 of the top 51 5-mers contain CGT/ACG (Figure 2 and Table S1). Surprisingly, neither GC content nor CpG island content is significantly different between the two sets of genes (unpublished data). Therefore, one might hypothesize that particular types of GC-rich sequences, such as those containing CGT/ACG motifs, are important for escaping X inactivation. Similarly, particular types of AT-rich sequences may be important for being subject to inactivation, rather than the overall GC content or association with CpG islands.

### SVM Classifiers Can Discriminate between Regions Escaping and Subject to X Inactivation

Although the distributions of the above features are significantly different in the genomic contexts of genes subject to or escaping inactivation, no single feature or even combination of two features is sufficient to accurately discriminate between the two classes of genes. We therefore investigated whether we could classify genes using linear SVM classifiers trained with large sequenced-based feature vectors.

Most genes in the XCR are subject to inactivation with few exceptions, while about half of the genes in the XAR escape inactivation [12]. Thus, the 110 genes of known and consistent X inactivation status in the XAR provide the most attractive dataset for classification experiments. To reduce computational complexity and based on the above statistical analyses, we only considered the 5,596 repeat, 3-mer, and 5-mer sequence features derived from 50-kb and 100-kb windows. To measure the accuracy of SVM classification, we performed cross-validation (CV) experiments using these 110 XAR genes (see Materials and Methods). Though many genes are less than 100 kb from each other, there is actually minimal overlap of sequence in common features, partially due to features being based on windows upstream and downstream separately. Nevertheless, to ensure that these overlaps were not biasing classification, we created groups of genes such that no feature from one gene overlapped a similar feature from another gene outside of its group by more than 10 kb. Using these groups and all 5,596 features, we classify 90/110 (82%) genes correctly in CV experiments (Table 2).

**Table 2:**
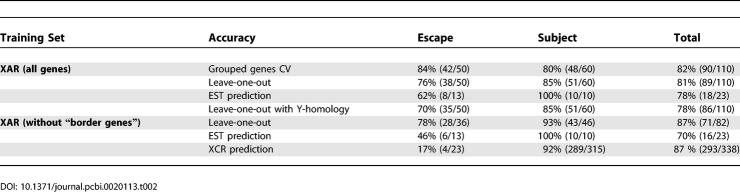
Classification Accuracy for XAR Genes, XAR ESTs, and XCR Genes Using 5,596 Features from 50-kb and 100-kb Windows around Transcription Start Sites of the Genes

Next, we performed leave-one-out CV experiments to determine whether there was a significant difference in accuracy. Using the same 5,596 features, the X inactivation status for 89/110 (81%) genes were correctly predicted, indicating that the overlaps in feature windows for nearby genes do not bias classification results (Table 2). Further, a model trained using these 110 genes achieves 78% classification accuracy on a completely independent set of 23 expressed sequence tags (ESTs) in the XAR for which the X inactivation status is known. Altogether, these results suggest that these sequence features do contain information that can be utilized for biological classification of both XAR genes and ESTs.

Since the majority of genes with functional Y-homologs escape inactivation [12], we created a set of independent features to capture this information and again performed leave-one-out classification to see whether accuracy improves. As shown in Table 2, classification results are nearly identical, suggesting that the Y homology status does not add additional information not already contained in other sequence features.

It has been noted previously that genes subject to or escaping inactivation are clustered regionally into domains [12]. Interestingly, 14 of the 21 misclassified genes in leave-one-out experiments are “border genes,” located within 100 kb of a gene from the other class as opposed to being internal to a cluster. Their incorrect classification may be due to the sequence window extending beyond the true escaping or subject domain, thus causing feature information from the other class to be associated with that gene. Excluding these border genes increased leave-one-out classification accuracy to 87% (Table 2) and is most likely a better assessment of the true accuracy of SVM classifiers trained on these 5,596 features.

To investigate whether the same signals may be used for X inactivation in the XCR, we used SVM models trained on XAR nonborder genes and predicted the X inactivation status for genes in the XCR. As shown in Table 2, SVM models can predict 87% XCR genes correctly overall, though they do not predict escaping genes well (four of 23 correct). Escaping genes are generally clustered in the XCR as in the XAR, albeit in smaller clusters, with 14 of the 23 genes contained within two clusters of three genes and two clusters of four genes. It is interesting to note that among the four escaping genes correctly predicted, one is a member of a four-gene cluster and the other three constitute one of the three-gene clusters. These results, therefore, suggest that the influence of sequence on the spread and/or maintenance of X inactivation is likely similar on the two evolutionarily distinct portions of the X chromosome.

### Feature Selection Identifies Informative Features Important for Classification

Based on our initial statistical analysis, it is likely that most sequence features do not contain discriminatory information that would contribute to accurate classification. Therefore, we systematically eliminated probable noninformative features to determine the affect on the classification accuracy of linear SVMs (Materials and Methods). In addition, we wanted to identify those features deemed by the SVM to be the most important for classification.

**Figure 3:**
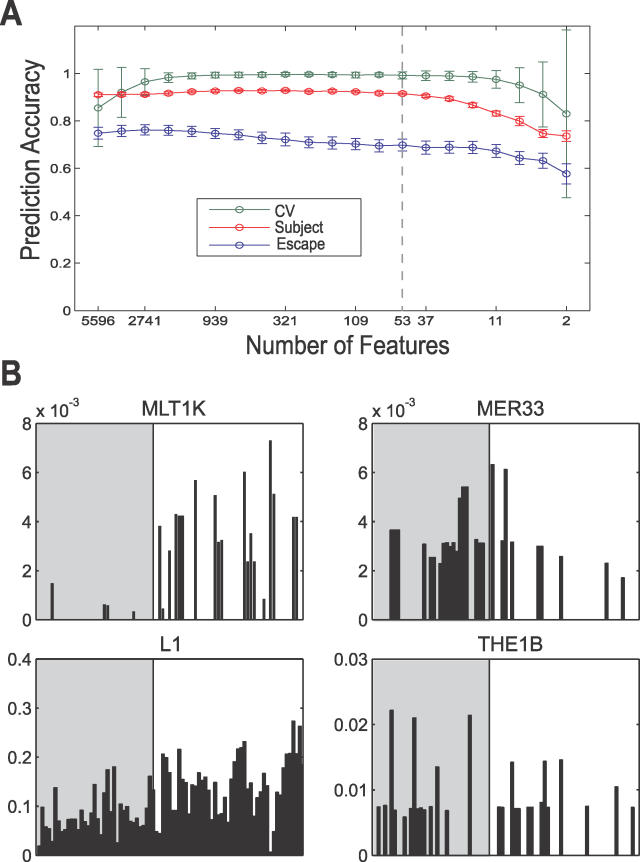
Recursive Feature Reduction and Distributions of Consistent Features across the XAR Nonborder Genes (A) The mean prediction accuracy and standard deviations (*y*-axis) for 100 recursive feature reduction iterations are shown for the indicated number of genes (*x*-axis). Green represents the CV rate using randomly selected two-thirds of the XAR nonborder genes for each set of features. The prediction rates for escaping genes (blue) and subject genes (red) in the remaining one-third are also shown. Both escape and subject prediction rates begin declining when the feature set is reduced to fewer than 53 features. (B) The content of each feature (*y*-axis) in specific windows around the transcription start sites for all 82 XAR nonborder genes (*x*-axis) is represented as a histogram. The first 36 genes on the *x*-axis escape X inactivation (shaded area), and the remaining 46 are subject to X inactivation. Features found to be consistently chosen during recursive feature reduction for the creation of accurate classifiers are L1 100 kb downstream, MLT1K 100 kb upstream, and MER33 100 kb upstream. For comparison, THE1B 50 kb upstream, a randomly distributed feature, is also shown.

In an effort to compile a robust set of informative features, this feature selection process was performed 100 times. In each iteration, a randomly selected set of two-thirds of the XAR nonborder genes was used. On average, reduced sets of 53 features performed as well in classification tests as when utilizing the full complement of features (Figure 3A and Table S3).

We identified features that appeared 20 or more times within these 100 reduced sets of 53 features (Table S4) with those most frequently selected (≥50) listed in Table 3. Many of these features also showed significant enrichment in regions around subject or escaping genes in the above statistical analysis (Table S1). More specifically, we find LINEs (L1 and L2) among the most informative features. In addition, short interspersed nuclear elements (MIR, MIRb, and FAM), LTRs (MLT1K, MLT1E2, ERV1, and LTR8), and DNA transposons (MER33 and MER112) are consistently selected, along with a 3-mer (CAC) motif and two 5-mers (CGCGC, AGTTG).

**Table 3:**
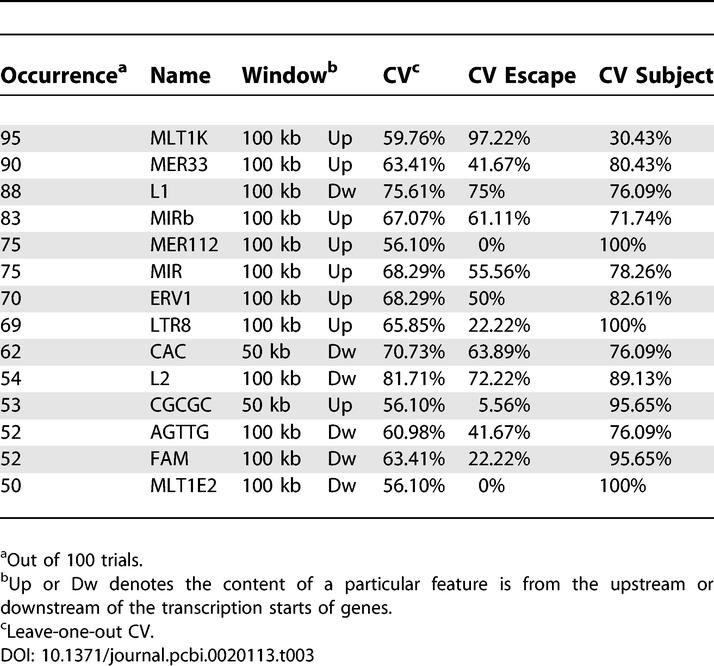
Frequently Selected Features during 100 Independent Feature Selection Experiments Involving Random Subsets of XAR Nonborder Genes and Their Individual Classification Performance on XAR Nonborder Genes

Informative features that contribute to accurate classification are generally those that are consistently lower (or higher) in one class as compared with the other. To explore why the most consistently selected features might be desirable for classification, we compared their content in the windows around each of the 82 XAR nonborder genes, a subset of which are shown in Figure 3B. We can see, for example, that L1s are present in 100-kb windows downstream of the transcription start site for all genes, but their content in these regions is typically higher in genes subject to inactivation. On the other hand, MLT1K is almost exclusively found in windows 100 kb upstream of genes subject to inactivation, while MER33 is primarily found only in windows 100 kb upstream of genes escaping inactivation. Although these MLT1K and MER33 features by themselves are poor classifiers overall (Table 3) and neither were found to have significantly different distributions in the two sets of genes based on our statistical analysis, their extreme bias toward genes in one class make them highly informative for classification. Most uninformative features, such as the content of THE1B 50 kb upstream of genes, are randomly distributed among all genes and thus are not good features for classification (Figure 3B).

### A Set of 12 Features Can Accurately Classify X Inactivation Status

**Figure 4:**
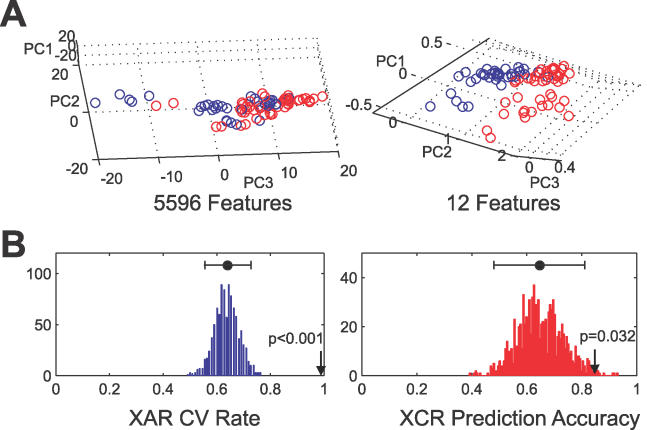
The Significance of the 12 Selected Features (A) The three best principal components (PC1–PC3) among all 5,596 features for 50-kb and 100-kb windows (left) and the selected 12 features (right) for the 82 nonborder genes are shown projected onto a 3-D graph. Escaping genes are represented as blue circles and subject genes as red circles. (B) These histograms show the distribution of XAR leave-one-out CV and XCR prediction rates by SVM models constructed using 1,000 random 12-feature sets taken from the 5,596 features for 50-kb and 100-kb windows. Black dots represent mean values, flanked by 95% confidence intervals denoted by error bars representing two standard deviations (2SD). Both the XAR CV rate and XCR prediction rate achieved by the selected 12 features (black arrows) exceed 2SD, and their *p* values calculated based on these random trials are shown.

To build an efficient classifier with an even smaller set of features, we repeated this feature reduction experiment with all XAR nonborder genes to produce a set of 17 features (Figure S1). This set was further refined using a combination of hierarchical clustering and principle component analysis (Materials and Methods). As a result, a SVM classifier constructed using a group of 12 features (Table S5) performed well on other datasets (Table 4), suggesting that these 12 features contain sufficient information for accurate classification. To visually demonstrate how these 12 features can separate genes in the two classes, we projected the three best principal components of this dataset into 3-D space and likewise did the same for the original 5,596 features (Figure 4A). Finally, these 12 features significantly outperform randomly selected 12-feature sets for both genes in the XAR training set and the XCR testing set (*p* < 0.001 and *p* < 0.05, respectively; Figure 4B). Therefore, these 12 features together form a representative set sufficient for accurate classification of X inactivation status.

**Table 4:**
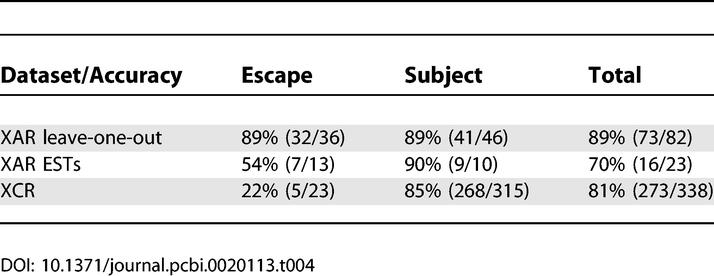
Classification Accuracy for XAR Genes, XAR ESTs, and XCR Genes Using a Reduced Set of 12 Features and XAR Nonborder Genes

### Confidence of SVM Classifiers

**Figure 5:**
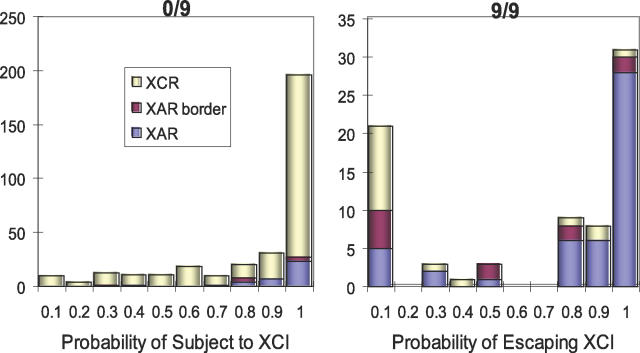
The Distribution of SVM Prediction Probabilities for Genes with Known X Inactivation Status These histograms summarize the prediction probabilities of genes that are either (A) subject to inactivation (expressed in zero of nine somatic cell hybrids) or (B) escape from inactivation (expressed in nine of nine hybrids) [12]. Genes from the XCR, XAR border genes, and nonborder XAR genes coupled with XAR ESTs are represented by different colors. XCI, X chromosome inactivation.

To assess the confidence of the SVM predictions, we output the probability for each prediction from LIBSVM for all genes on the X-chromosome (Table S6). The SVM model used for this analysis was trained using the 12-feature set for the 82 nonborder genes. In the comprehensive X inactivation survey [12], expression of genes from the inactive X chromosome in mouse/human hybrid cell lines was divided into ten categories based on how many cell lines show a particular gene escaping X inactivation. Most of the correctly predicted genes had probabilities higher than 0.75 (unpublished data), indicating high confidence in these predictions, while probabilities for wrongly predicted genes are evenly distributed showing greater uncertainty. For genes of consistent X inactivation status (i.e., those subject to inactivation in all hybrids tested; *n* = 324), most were predicted correctly with high confidence (>0.9 in Figure 5). Similarly, for the smaller number of genes that escape inactivation in all hybrids (*n* = 76), the majority were correctly predicted with probabilities >0.8 (Figure 5). Notably, however, a subset of such genes was incorrectly predicted to be subject to inactivation with high probability (Figure 5). Detailed analyses revealed most of these genes are either XAR border genes or genes from the XCR. This suggests that there may be features associated with genes that escape inactivation, especially in the ancestral XCR, that have not been revealed in this analysis based on XAR genes.

A University of California Santa Cruz (UCSC) Browser annotation track has been created using expression and prediction information to facilitate comparison with other genome annotations (Figure S3). This is available at http://genome-mirror.duhs.duke.edu a full mirror site maintained within the Institute for Genome Sciences and Policy (IGSP) at Duke University.

## Discussion

We have shown that the genomic environments of genes subject to and escaping X chromosome inactivation are significantly different, especially with respect to the content of LINEs, MIRs, Alu elements, and short sequence motifs containing the three-nucleotide sequence CGT/ACG. Based solely on primary DNA sequence, linear SVM classifiers can correctly predict 80% of all the genes and 87% of genes located in the interior of clusters of genes sharing the same X inactivation status. These results suggest that most, if not all, of the information necessary to determine X inactivation status is embedded in primary DNA sequence. Furthermore, our analyses indicate that this information can be represented by as few as 12 sequence features.

### Being Subject to or Escaping from X Inactivation Is Likely Dependent on Several Factors

Lyon proposed that L1 elements could serve as “booster elements” for spreading of X inactivation [20]. Multiple copies of transgenes or transposons can induce heterochromatin formation and gene silencing in flies and mammals [31,32]; therefore, chromosomal regions enriched for L1 elements may also promote spreading of inactivation. Conversely, regions deficient in L1 elements may lack the ability to promote X inactivation, thus allowing genes located within such regions to at least partially escape silencing. Consistently, L1 and L2 elements are among the most important features for accurate SVM classification (Tables 3 and S4), and classifiers created using these features alone have a prediction accuracy of about 77% in leave-one-out CV experiments.

However, LINE elements alone are not sufficient to correctly classify all samples, indicating that this feature by itself does not determine the X inactivation status of a region. For example, another frequently selected feature, MLT1K, classifies the escaping gene class almost perfectly due to its being found nearly exclusively around subject genes (Figure 3B) but has poor classification accuracy on subject genes (Table 3). In addition, other sequence features may prevent spreading by disrupting at least some feature of facultative heterochromatin formation [18], as evidenced by their positive correlation with regions containing escaping genes. Therefore, efficient spreading and/or maintenance are likely affected by both the presence and absence of certain sequence features.

### Do Alu Elements Play a Role in Escaping from X Inactivation?

Alu repeats are primate specific and represent more than 10% of the human genome [33]. They are GC rich and contain one-third of all CpG islands. Alu repeats have been implicated in many biological processes, including DNA recombination, DNA methylation, and gene expression regulation (reviewed in [34]). Alu elements have been shown to be excluded from imprinted regions and are differentially methylated in male and female germ lines, suggesting a potential influence on imprinting [35–37], a process that may have coevolved with X inactivation [38]. The relatively low proportion of genes escaping X inactivation in the mouse may be consistent with the much lower B1 (the Alu equivalent) content in the mouse X-chromosome (~2%). Combined with the correlation of Alu content with escaping genes on the human X that we demonstrated in our statistical analysis, these data seems to indicate a potential role of Alu elements in facilitating a gene's escape from X inactivation. However, results from our classification experiments suggest that Alu sequence features are not as informative for classification as other features. Although Alu content is very high in some genes that escape inactivation (mostly from strata 4–5), their distribution is highly variable among of the rest of XAR genes (Figure 2). This may explain why Alu features do not discriminate as well as other features. Furthermore, Alu features are not among the top of those consistently selected in reduced feature sets. This seems to argue against an important role for Alu elements in influencing X inactivation status.

### A Set of 12 Sequence Features Predicts X Inactivation Status

Through systematic feature reduction, we obtained a set of 12 features that alone contain sufficient information for accurate classification. It seems reasonable to hypothesize that some of these features may act as signals for some aspect of X inactivation. However, the feature reduction method we used does not guarantee an optimal final set of features. We can find six alternative 17-feature sets that achieve comparable classification rates as the 12-feature set (Table S3). Therefore, some features within this 12-feature set may not be necessary for determining the X inactivation status, while other important features may be excluded. A more robust set of features were compiled through multiple feature selection tests (Table 3), but again this does not show without a doubt the involvement of these specific features in X inactivation. Further analyses—both computational and experimental—will be essential to determine what role, if any, these specific features have in the X inactivation process.

### Some Genes in the XCR Might Employ Different Mechanisms for Escaping Inactivation

SVM classifiers trained using genes from the XAR are able to correctly predict the status of 87% of XCR genes that are subject to X inactivation using all features from 50-kb and 100-kb windows (Table 2), and 81% of XCR genes using the reduced feature set (Table 4). This suggests that X inactivation uses a similar mechanism to silence genes in the XCR as in the XAR. In contrast, most escaping genes in the XCR are incorrectly predicted to be subject to X inactivation (Tables 2 and 4). Encouragingly, SVMs successfully predicted at least part of two of the four clusters of escaping genes on the XCR, possibly indicating a similar mechanism to escaping genes that are clustered on the XAR. One of the other clusters of escaping genes on the XCR that SVMs completely fail to predict correctly includes *XIST* itself, and this region may require a different mechanism to escape X inactivation. The other misclassified cluster was recently shown to be flanked by CCCTC-binding factor sites that may serve as “boundaries” to spreading of inactivation [39]. Since CCTC-binding factor sites are not well defined currently, it is unknown whether other escaping genes in the XCR may be flanked by such putative boundary elements. It is quite possible that other genes on the XCR escape inactivation through mechanisms very different from those that facilitate escape in the XAR due to different evolutionary pressures and histories.

### A Possible Role for Genomic Sequence in X Inactivation

The exact relationship between primary DNA sequence and X chromosome inactivation is still not known. While the SVM classifiers created using the sequence features we selected are able to predict X inactivation status well, especially when only considering genes internal to clusters, they are not perfect. The most likely reasons for this include: (1) DNA sequence alone does not contain sufficient information to definitively determine X inactivation status; (2) the features identified in this study do not adequately characterize the sequence characteristics that determine X inactivation status; or (3) higher-order structures of chromosome or chromatin packaging provide long-range signals that are not adequately captured by consideration of relatively short-range windows of sequence in the vicinity of each gene, as performed here. For example, the repeat sequences and the 3-mers and 5-mers may not directly reflect structural properties of the underlying DNA that may facilitate or impede silencing. Further, it has been shown recently that the structural properties of DNA are involved in the positioning of nucleosomes on the genomic sequence, which in turn influences heterochromatin formation (reviewed in [40]).

We propose that structural characteristics underlying DNA in certain regions of the X chromosome may prevent the necessary chromatin packaging required to suppress gene expression. Thus, a sequence that is not amenable to high-density nucleosome array formation could interfere with *XIST* RNA spreading and/or the ability to maintain a silenced state. Even without positing a specific role for the repeat elements directly, the base composition of certain repetitive elements such as Alu elements and L1s may contribute (L1s) or prevent (Alu) the proper packaging necessary for optimal spread and/or maintenance of inactivation. Their abundance on the X-chromosome could allow them to act as good proxies for this information, and thus allow the creation of accurate classifiers.

Further study into the structural properties of chromosomal DNA and their relationship to both repeat sequences and X inactivation should allow for the evaluation of higher-order models containing potentially more informative aspects of the relevant sequence features. Such features may enable us to completely discriminate between regions that escape or are subject to X inactivation.

## Materials and Methods

### Genes and ESTs.

Genes and ESTs (transcripts) considered for this analysis consist of those for which the X inactivation status is known, as previously described [12,41]. For classification experiments, we designated transcripts as “escape” or “subject” on the basis of criteria established previously [12]. Briefly, escaping transcripts are those showing in expression in at least 75% of the hybrids, while subject transcripts are silenced in at least 75% of the hybrids, with results being obtained for at least five hybrids. In addition, transcripts must be mapped to a single location in the Human Genome build 35 (hg17). Of 621 uniquely mapped transcripts, 561 meet the above criteria.

The XAR is defined precisely as the first 46,700,000 bases in the build 35 assembly of the X chromosome. According to these criteria, there are 50 annotated genes in the XAR that escape inactivation and 60 that are subject to inactivation. In addition, there are 20 ESTs that escape and 18 ESTs subject to inactivation in this region. Of these 38 ESTs, 15 overlap transcribed regions of genes in the XAR and were discarded, leaving 13 that escape and ten that are subject. In the XCR, the above criteria identified 23 escape genes and 315 subject genes.

Gene groups were created by calculating the overlap of every genes' 100-kb upstream (downstream) regions with neighboring genes' upstream (downstream) regions. Genes with an overlap exceeding 10 kb were placed in the same group. In a particular group, a gene did not necessarily overlap all of the other genes, just at least one other gene with respect to upstream or downstream sequence. A total of 62 groups were created for the 110 XAR genes.

### Sequence features.

Content of 310 repeat families and subfamilies as annotated by RepeatMasker (http://www.repeatmasker.org) and defined in Repbase (http://www.girinst.org), CpG islands, as well as of all 64 three-base and 1,024 five-base sequences, were extracted from the X chromosome sequence [15] in 2-, 5-, 10-, 20-, 50-, and 100-kb windows from both upstream and downstream of the transcription start site of each gene, yielding a total 12 windows. (A few rare types of repetitive elements that occur fewer than ten times on the X chromosome were eliminated from consideration due to a lack of power.) This resulted in a total of 16,788 individual features. For ESTs, the 3′-ends were arbitrarily treated as their transcription start sites, because neither their transcript starts nor their transcription direction is known. All sequence information is based on the National Center for Biotechnology Information (NCBI) Human Genome build 35 assembly and was obtained from the UCSC Genome Browser (http://genome.ucsc.edu).

### Y-homology features.

A vector of three binary values was created to differentiate and characterize the presence of homologous transcripts on the Y chromosome for each gene. These vectors are defined as the following for four different classes of genes: pseudoautosomal genes (1, 1, 1), genes with functioning Y-homologs (0, 1, 1), genes with Y-linked pseudogenes (0, 0, 1), and genes with no apparent Y-homolog or pseudogene (0, 0, 0).

Our final dataset was a matrix with features as columns and genes as rows. In addition, the X inactivation status of each gene is represented as 1 for escaping and −1 for being subject to inactivation.

### Modified Wilcoxon rank-sum test and *q* values for false discovery rate.

Statistical tests are based on the Wilcoxon rank-sum test/Mann-Whitney *U* test. Features with zero values for all genes were excluded from all analyses. For each feature, we defined a weight *W* to characterize the difference in the distribution of that feature in regions of escaping and subject genes. Specifically, we defined *W* as follows:

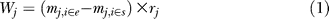
where *m_j,i∈e_* is the median rank for the escaping genes, *m_j,i∈s_* is the median rank for the subject genes, and *r_j_* is the Pearson correlation of the *j*th feature to X inactivation status. To assess the significance of *W* for a particular feature, a *p* value was calculated by randomly permuting gene labels 1,000 times and calculating a weight for each permutation. The null hypothesis was that the two samples (escaping and subject genes) are drawn from the same population. The *p* value assigned reflects the percentage of permutations whose weight was greater than the original *W* and reflects the probability that a *W* value of this magnitude was obtained by chance. To assess better the significance of these *p* values, *q* values were calculated and provide a measure of the false discovery rate (see [30] for a more complete description of this calculation). All analyses were carried out using Matlab7 R14 software (The Mathworks, Natick, Massachusetts, United States). *q* values were calculated using the software QVALUE v1.0 [30].

### SVM classification and recursive feature selection.

LIBSVM version 2.71 software was used for SVM classification (http://www.csie.ntu.edu.tw/~cjlin/libsvm). SVM training, prediction, weight calculation, and probability calculation were performed based on instructions that accompany the software. Namely, the sparse format was used, and the *C* value was dynamically calculated for each individual test based on the training data. Feature values were scaled for each experiment such that for each feature, values ranged between 0 and 1.

Each gene or EST was represented by a feature vector consisting of real valued numbers representing the sequence features described previously. In addition, each gene or EST was labeled as either escaping or being subject to X inactivation. For a given set of genes, CV experiments were performed as follows: in an iterative fashion, linear SVM models were trained on all the genes or all groups of genes except one. The resulting SVM classifier was used to predict the inactivation status (i.e., subject or escape) of the held-out gene or all genes in the held-out group. Prediction accuracy was calculated based on results for all the genes in the set.

Recursive feature selection was carried out as follows: starting with 5,596 sequence features from 50-kb and 100-kb windows, linear SVM classifiers were trained using a given set of genes. Features were sorted based on their weights in the resulting SVM classifier, and those whose weight was in the lowest 30th percentile were eliminated. This procedure was repeated in an iterative fashion to generate progressively smaller feature sets until the number of remaining features was less than ten. Accuracy of classification was assessed at each step by performing both leave-one-out CV on nonborder genes from the XAR and prediction of X inactivation status of genes from the XCR.

### Hierarchical clustering.

For the reduced set of 17 features for the 82 XAR nonborder genes (see Results), values for each feature were normalized such that the mean value was zero and the standard deviation was one. Hierarchical clustering was performed using the following formula in Matlab:

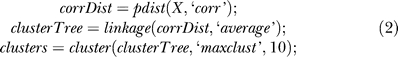
where *X* is the normalized matrix with the features as rows and genes as columns. The dendrogram in Figure S2A was drawn with the color threshold set to 0.7.

### Principal component analysis.

Feature vectors consisting of all 5,596 features from 50-kb and 100-kb windows and, separately, the reduced set of 12 features for the 82 XAR nonborder genes were normalized as described above. For each dataset, the top three principal components were calculated. Finally, the values for these three principal components for the 82 nonborder genes were projected onto a 3-D graph to visualize their separation.

### Reduced feature set significance.

To determine the significance of the 12 selected features, 1,000 random sets of 12 features were drawn with replacement from the complete set of 5,596 features from 50-kb and 100-kb windows. The null hypothesis being tested was that the 12 selected features are randomly selected and therefore do not perform better than other randomly selected feature sets. Leave-one-out CV experiments were performed as described above for each of the random sets, and their prediction accuracies were determined.

## Supporting Information

Figure S1 Recursive Feature Reduction on All XAR Nonborder Genes The top plot represents the CV rate on XAR nonborder genes for each set of features. The bottom plot represents the prediction rate for XCR genes using SVM models constructed from XAR nonborder genes. At 17 features, both CV and prediction rates reach their respective maximum values.
(90 KB PDF)

Figure S2 The Hierarchical Clustering of 17 Features Features are represented by numbers; labels of *x*-axis in (A) and numbers in parentheses in (B):
1, MLT1K 100 kb downstream (dw); 2, L1 100 kb dw; 3, AGGCA 50 kb upstream (up); 4, L2 100 kb dw; 5, MLT1K 100 kb up; 6, CGGTG 100 kb dw; 7, MIRb 100 kb up; 8, ATAGG 50 kb dw; 9, Charlie1 100 kb dw; 10, MER20 100 kb up; 11, TGACT 50 kb dw; 12, MLT2B3 100 kb dw; 13, ACCCC 50 kb dw; 14, MER3 50 kb up; 15, TCTGC 100 kb dw; 16, CTCAT 50 kb up; 17, GTTTG 50 kb up.
(A) A dendrogram of ten hierarchical clusters. Features whose edges have the same color belong to the same cluster.
(B) Plots showing the distribution of features within each cluster for 82 XAR nonborder genes.
(443 KB PDF)

Figure S3 UCSC Genome Browser Track for X Inactivation Status and Prediction of All X-Linked Genes Genes and ESTs are denoted by solid brown bars. The color intensity reflects the propensity for escaping inactivation, with the darkest brown indicating those that escape in all experiments (based on somatic cell hybrid data in [12]). Detailed information about the number of hybrids tested and results from SVM predictions can be seen by clicking on individual elements in this track.
(23 KB PDF)

Table S1 Sequence Features Found To Be Statistically Different between Escaping and Subject Genes for Whole X Chromosome (ALL, *q* < 0.05), XAR (*q* < 0.05), and Stratum 3 (S3, *q* < 0.2), Respectively (574 KB XLS)

Table S2 Subset of Sequence Features Found To Be Statistically Different between Escaping and Subject Genes Commonly in Each of the Whole X Chromosome (ALL), XAR, and Stratum 3 (S3) (50 KB XLS)

Table S3 CV and Prediction Rates for SVM Classification Experiments Using 100 Sets of Genes Randomly Drawn from XAR Nonborder Genes (40 KB XLS)

Table S4 Frequently Selected Features during 100 Independent Feature Selection Experiments Involving Random Subsets of XAR Nonborder Genes (21 KB XLS)

Table S5 Leave-One-Out CV rates for XAR Nonborder Genes and Prediction Accuracy for XCR Genes of SVM Classifiers during Recursive Feature Selection (18 KB XLS)

Table S6 The Predicted X Inactivation Status of All X-Linked Genes along with the Associated Probability of Prediction as Determined by a SVM Classifier Constructed Using a Reduced Set of 12 Sequence Features (86 KB XLS)

## Abbreviations

CV: cross validation
EST: expressed sequence tag
LINE: long interspersed nuclear element
LTR: long terminal repeat
MIR: mammalian-wide interspersed repeat
SVM: support vector machine
XAR: X-added region
XCR: X-conserved region
