# Supplementary material for: Evidence of Influence of Genomic DNA Sequence on Human X Chromosome Inactivation
Source: PLoS Comput Biol. 2006 Sep 1;2(9):e113. doi: 10.1371/journal.pcbi.0020113 (PMC1557588; doi:10.1371/journal.pcbi.0020113)
Supplement: Supplementary file 2 [file pcbi.0020113.sg002.pdf]

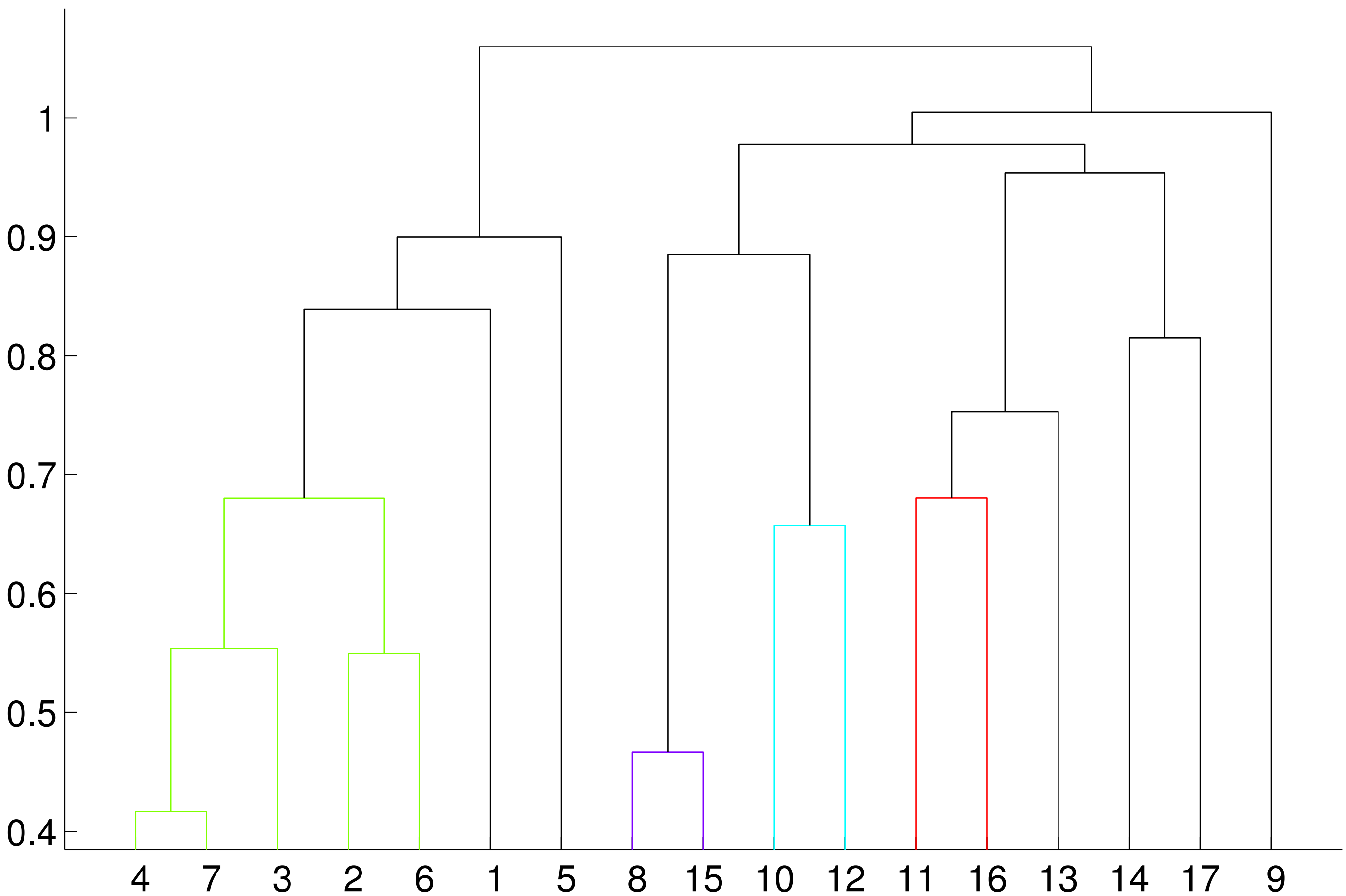

Figure S2A. Wang et al., 2006

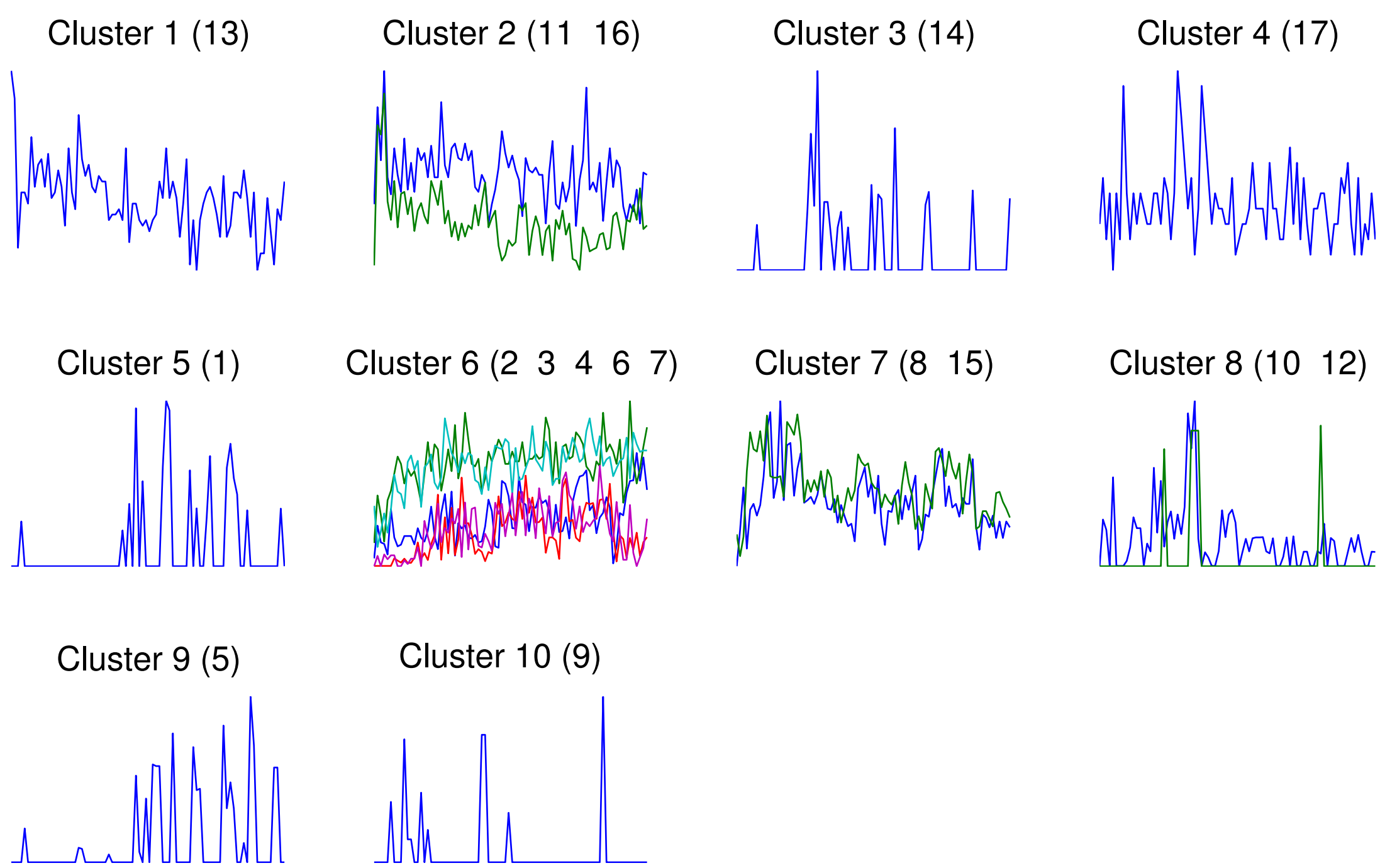

Figure S2B. Wang et al., 2006
