# Supplementary material for: Evidence of Influence of Genomic DNA Sequence on Human X Chromosome Inactivation
Source: PLoS Comput Biol. 2006 Sep 1;2(9):e113. doi: 10.1371/journal.pcbi.0020113 (PMC1557588; doi:10.1371/journal.pcbi.0020113)
Supplement: Supplementary file 3 [file pcbi.0020113.sg003.pdf]

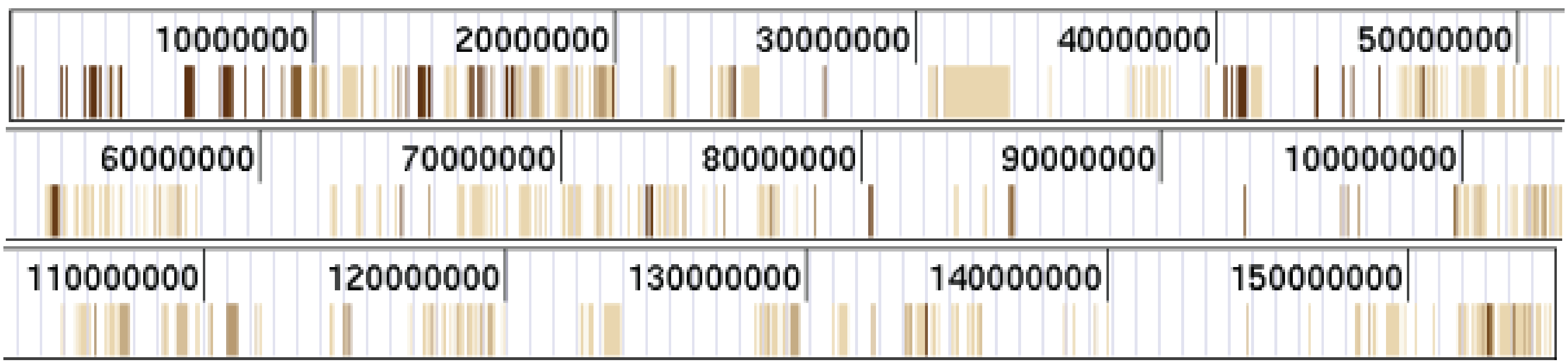

Figure S1. Want et al. 2006
